# Regulation Progresses of Selenium Improving Intestinal and Extra-Intestinal Tissues Health Through Regulating Gut Microbiota

**DOI:** 10.3390/biology15110887

**Published:** 2026-06-04

**Authors:** Yanle Fan, Wenjun Zhang, Wenjing Zhuang, Xia Zhao, Yun Hu, Tingting Li, Xiaoyan Cui, Chuanlong Wang, Liyang Zhang, Xugang Luo, Shengchen Wang

**Affiliations:** 1 College of Animal Science and Technology, Yangzhou University, Yangzhou 225000, China; 2 College of Agricultural Science and Engineering, Liaocheng University, Liaocheng 252000, China; 3 Mineral Nutrition Research Division, State Key Laboratory of Animal Nutrition, Institute of Animal Science, Chinese Academy of Agricultural Sciences, Beijing 100193, China

**Keywords:** Selenium, microbiota, metabolism, intestinal health, gut-tissue axis

## Abstract

Selenium (Se) is an essential trace element that exerts pleiotropic effects on host physiology, yet the mechanisms by which it coordinates systemic health remain incompletely understood. Emerging evidence regards the gut microbiota as a key mediator of Se biological functions, giving rise to the Se–gut–tissue axis. This review synthesizes the current research progresses on how dietary Se may shape gut microbial composition and metabolism, and how these microbial shifts are associated with protective effects in both intestinal and extra-intestinal tissues. Se sources (particularly organic or new synthetic form) may bidirectionally interact with gut bacteria by enriching beneficial genera such as *Akkermansia*, *Lactobacillus*, and butyrate-producing *Clostridia*, while suppressing opportunistic pathogens. This microbial remodeling strengthens intestinal barrier integrity, enhances antioxidant and anti-inflammatory responses (e.g., via GPX, TrxR, and NF-κB suppression), and generates bioactive metabolites, notably short-chain fatty acids and secondary bile acids. Through these mechanisms, the Se–gut–microbiota axis may regulate distal organ homeostasis, including the liver (ameliorating NAFLD and acute injury), brain (counteracting neurodegeneration and modulating serotonin/GABA), muscle (improving mass and Se deposition), kidney (attenuating uremic toxin-induced ferroptosis), and reproductive organs. Despite encouraging progress, challenges remain in establishing causality, optimizing dose–response relationships, and translating findings into precision interventions.

## Simple Summary

Selenium is an essential trace element with multiple biological functions. However, previous research has mostly focused on its direct effects. The latest research has found that selenium may indirectly affect tissue health by regulating microbial composition and metabolism. This review summarizes the recent research findings to expand our understanding of the biological role of dietary selenium supplementation and proposes potential application challenges.

## 1. Introduction

Selenium (Se) is an essential micronutrient that exerts crucial physiological roles in mammals and a wide range of other organisms, from single-celled microbes to complex multicellular species [1]. Our understanding of the significance of the element in biological systems has developed dramatically over time. Historically, Se was primarily recognized for its toxic properties, with early studies focusing on its detrimental effects on livestock and wildlife in regions with high environmental Se concentrations [2]. However, this perception underwent a transformative shift following Klaus Schwarz’s seminal work in 1957, which definitively identified Se as a vital element necessary for normal physiological function [1,3,4]. This breakthrough discovery laid a theoretical foundation for studying its biological roles. Over the subsequent six decades, extensive research has revealed the multifaceted functions of Se that extend far beyond its initial association with disease prevention. These functions include central roles in antioxidant defense, where Se-containing enzymes such as glutathione peroxidases (GPXs) neutralize harmful reactive oxygen species [5,6], immune regulation, through the modulation of cytokine production and immune cell activity [7], and thyroid hormone metabolism, a process mediated by selenoproteins that govern the conversion of thyroid hormones into their biologically active forms [8]. Each of these roles highlights the indispensable status of Se biofunctions in maintaining systemic homeostasis. While Se is indispensable for systemic homeostasis, it exhibits a classic U-shaped dose–response relationship. Se deficiency compromises immune function, increases the risk of Keshan disease, and disrupts thyroid hormone metabolism, whereas excess causes selenosis (hair loss, nail deformation, gastrointestinal and neurological damage) and has been linked to the risk of metabolic disorder, such as sinsulin resistance and NAFLD, cautioning against one-sided optimism [9,10].

Research into the gut microbiota, an immense and highly diverse community of microorganisms that colonize the intestinal tract, has revealed its profound impact on host physiology. Comprising predominantly bacteria, along with archaea, fungi, and viruses, this microbial ecosystem establishes a complex symbiotic relationship with the host [11]. Through this symbiosis, the gut microbiota contributes to many critical functions, such as the breakdown and absorption of nutrients that the host cannot digest independently [12], regulation of immune system development and activity, maintenance of the intestinal barrier integrity to prevent pathogenic invasion [13], and even the exertion of far-reaching effects on the health of extra-intestinal tissues by modulating metabolic pathways and coordinating interorgan signaling networks [14]. The intricate interplay between the gut microbiota and the host has thus emerged as a key area of study in understanding health and disease. Nevertheless, when this delicate microbial balance is disrupted, a state known as dysbiosis, the consequences for host health can be significant [15,16].

Se executes biological functions mainly through synthesizing selenoproteins. Up to now, 21 selenoproteins have been found in vertebrates [17]. Key selenoproteins include GPXs (GPX1-4), thioredoxin reductases (TrxR1-3), selenoprotein P (SELENOP), SEP15, SELK, SELS, and SELM. GPXs and TrxRs family members are major antioxidant enzymes to maintain intracellular redox balance. SELENOP serves as the primary Se transporter in plasma. Endoplasmic reticulum-resident selenoproteins (SEP15, SELK, SELS, SELM) participate in protein folding and alleviate endoplasmic reticulum stress. Therefore, the body must maintain sufficient levels of Se to ensure appropriate expression of selenoproteins in various physiological and pathological stages. Recent evidence highlights the bidirectional interplay between dietary Se and gut microbiota: dietary Se modulates gut microbial composition, diversity, and colonization, while these shifts influence host Se status (absorption, distribution, and utilization) and selenoprotein expression [18]. Notably, genomic studies show approximately 25% of bacterial species, including gut colonizers like *Escherichia coli*, *Clostridium difficile*, and some members of *Enterobacteriaceae*, harbor selenoprotein-encoding genes [19]. These microbial communities actively synthesize selenoproteins for their metabolism, potentially affecting host Se bioavailability and acting as active participants rather than passive recipients. Advancing research indicates that dietary Se supplementation, gut microbiota, and their metabolites coordinate animal tissue health via complex pathways involving microbial metabolites, host gene regulation, immune/antioxidant modulation, and other biological processes, forming an emerging axis linking Se intake to multi-organ homeostasis. This review examines how dietary Se may reshape gut microbiota and metabolites, thereby influencing both intestinal and systemic tissue homeostasis. It is worth noting that most of these relationships are not a unified linear regulatory system, but highly dependent on partial correlations of the model, which may vary in different types of research. By dissecting key signaling pathways within this interplay, we elucidate potential or possible molecular mechanisms by which Se drives tissue health. Our analysis transcends traditional nutritional concepts that only focus on the direct biological effects of Se intake, providing innovative perspectives for guiding human disease management strategies and even optimizing animal production practices.

## 2. Se Metabolism and Gut Microbiota Crosstalk

Host Se metabolism begins with dietary intake of both organic forms (e.g., selenocysteine [Sec] and selenomethionine [SeMet]), inorganic forms (e.g., selenite [SeO_3_^2−^] and selenate [SeO_4_^2−^] salts), a novel nano form (nano-Se), and Se-enriched probiotics or polysaccharides [6]. Substantial evidence indicates that organically bound Se sources and nano-Se exhibit higher bioavailability compared to inorganic Se compounds [20,21]. This distinction arises from the fact that organic Se is efficiently absorbed via amino acid transporters (e.g., solute carrier family 7 member 11 [SLC7A11]) localized on intestinal epithelial cells, nano-Se is absorbed via efficient endocytosis pathway, while the uptake of Se from SeO_4_^2−^ and SeO_3_^2−^ is presumed to be realized through Na^+^-dependent active transportation and passive diffusion [21,22,23]. Following gastrointestinal absorption, in addition to the endocytic nano-Se particles forming vesicles inside cells, which are processed and released into the extracellular space through the basal membrane, other Se forms need to be metabolized in intestinal epithelial cells and then transported to the liver and other tissues through the portal vein circulation for utilization [24]. The metabolic pathways intestinal tract vary depending on the Se form. Inorganic Se undergoes stepwise reduction: SeO_3_^2−^ (partially converted from SeO_4_^2−^) in intestinal epithelial cells first reacts with glutathione (GSH) to form selenodiglutathione (GS-Se-SG). This intermediate is then reduced by glutathione reductase to generate glutathioselenol (GS-SeH), which ultimately releases hydrogen selenide (H_2_Se); organic Se enters the trans-selenation pathway: SeMet is first metabolized to selenohomocysteine (SeHcy) by methionine adenosyltransferase [25]. Subsequently, cystathionine enzymes catalyze its conversion to Sec, which is then cleaved by selenocysteine lyase to release H_2_Se. Finally, H_2_Se generated from the conversion of two types of Se sources can be converted into selenophosphate and then incorporated into nascent polypeptides at UGA codons guided by selenocysteine insertion sequence (SECIS) elements, to synthesize selenoproteins [26]. In addition, SeMet can either be directly utilized for the synthesis of selenoenzymes or nonspecifically substitute for methionine in general proteins, which effectively elevates whole-body selenium reserves [27]. Excessive Se is primarily methylated to trimethylselenonium, which is excreted in urine; small amounts of volatile dimethyl selenide (DMSe) are also eliminated via respiration [26].

**Figure 1:**
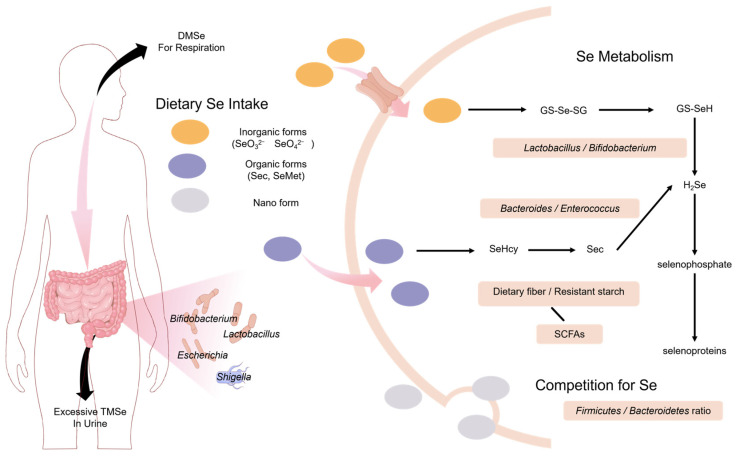
Mechanisms of reciprocal regulation of selenium metabolism, absorption, and bioavailability by gut microbiota and the host.

Notably, accumulating experimental evidence demonstrates that the gut microbiota exerts significant impacts on Se metabolism, with a range of diverse mechanisms collectively shaping host Se homeostasis (Figure 1). Gut microbiota drive Se speciation via a set of key enzymes, each playing a distinct role in transforming Se compounds. For instance, certain bacterial species within the *Bacteroides* (e.g., Bacteroides distasonis and Bacteroides vulgatus) and *Enterococcus* (e.g., *Enterococcus faecalis*, and *Enterococcus faecium*) exhibit higher activity of selenocysteine β-lyase, which participates in the decomposition of Sec to release HSe^−^ for the synthesis of selenoproteins [28,29]. Some probiotics (e.g., *Lactobacillus* and *Bifidobacterium*) can convert SeMet and SeO_3_^2−^ into dimethyl diselenide (DMDSe) and DMSe for excretion with the help of methyltransferase, or reduce SeO_3_^2+^ to H_2_Se through reductase, and then polymerizes into Se nanoparticles (100 to 400 nm) for absorption [30,31,32]. Microbial metabolites, particularly polyunsaturated fatty acids (PUFAs) and short-chain fatty acids (SCFAs) also contribute significantly to Se metabolism. For instance, docosahexaenoic acid, a type of PUFAs, could indirectly reduce Se absorption efficiency by inhibiting SLC7A11 expression and reducing cysteine uptake [33]. Meanwhile SCFAs, the products of dietary fiber and resistant starch metabolism by gut microbiota, can increase the solubility of SeO_4_^2−^ and help maintain the reduced form of Se (Se^2−^) by lowering the intestinal pH, thereby creating a more favorable environment for Se absorption [26]. However, despite the symbiotic relationship between the animal host and its gut microbiota, wherein the microbiota contributes to digestive processes and the host provides a nutrient-rich niche, competition for Se may arise under conditions of this micronutrient limitation [34]. This competition can impact the host’s Se status, as the microbiota may prioritize Se for their own metabolic needs, potentially limiting its availability for selenoprotein synthesis. Conversely, Se supplementation can reshape the gut microbial ecology: it may reduce the abundance of *Escherichia-Shigella*, which are associated with Se retention, while increasing populations of probiotics like *Lactobacillus* and *Bifidobacterium*. Additionally, it elevates the *Firmicutes*/*Bacteroidetes* (F/B) ratio, a shift that may have implications for overall gut function and metabolic health [35]. However, the reproducibility of the F/B ratio is poor and it is easily influenced by diet and environment; therefore, it is not sufficient to be fully considered as direct evidence for Se promoting gut microbiota homeostasis.

## 3. Se–Gut Microbiota Axis in Gut Health

Optimal gut health is defined as a state of dynamic homeostasis within the gastrointestinal system, characterized by integrated structural integrity, physiological functionality, microbial equilibrium, and systemic crosstalk [36]. Key features include efficient nutrient assimilation, robust mucosal barrier integrity, balanced microbial community, and regulated immune-inflammatory responses. Substantial evidence reveals that dietary Se intake plays a crucial role in remodeling gut microbiota to enhance intestinal barrier function and immunity. Se deficiency, with a dietary level as low as 0.01 mg/kg, has been found to increase the relative abundance of opportunistic pathogens taxa including *Salmonella typhi*, *Faecalibaculum*, *Helicobacter*, and *Odoribacter* in mice, which are closely associated with irritable bowel syndrome and ulcerative colitis, common disorders that affect a significant portion of the global population [37,38]. Meanwhile, Se deficiency-induced intestinal dysbiosis leads to a reduction in tight junction (TJ) proteins, such as ZO-1 and occludin. Under Se-deficient conditions, the expression levels of these TJ proteins can be reduced by up to 50% in animal models [39,40,41,42,43]. These proteins are fundamental in establishing a mechanical barrier in the intestinal epithelium, not only selectively regulating paracellular transport, but also preventing the infiltration of multiple pathogens and toxins [44]. In contrast, adequate Se intake, typically around 0.15 mg/kg, or even higher levels such as 0.40 mg/kg in mice, enriched the gut microbiota with beneficial genus, such *Turicibacter*, *Butyricimonas*, *Prevotella*, and *Akkermansia*, whose functions are beneficial under specific dietary and pathological circumstances [38,45,46]. These enriched microbial taxa have been shown to be associated with increased production of anti-inflammatory factors such as IL-10 and IL-22, thereby alleviating intestinal inflammation and indirectly maintaining TJ protein expression [47,48,49,50,51]. Specifically, the effects of different dietary Se on gut microbiota composition also vary to some extent. Inorganic Se shows low bioavailability and may cause fluctuating changes in the *Firmicutes*/*Bacteroidetes* ratio [52]. Organic Se has high biocompatibility and tends to enrich *Lactobacillus*, *Bifidobacterium*, and butyrate-producing bacteria [45]. Nano-Se exhibits high efficiency in enhancing *Akkermansia* and *muribaculaceae* [53]. Se-enriched probiotics and Se polysaccharides can synergistically regulate microbial homeostasis. These differences lead to form-dependent effects on host health.

**Figure 2:**
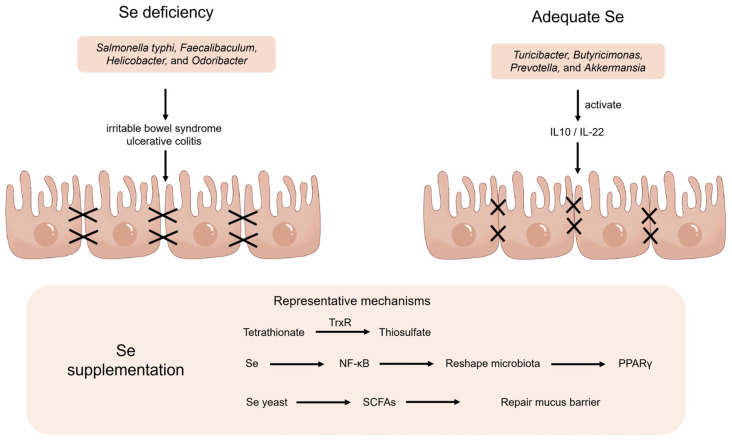
Mechanisms of selenium-supported gut health via microbiota remodeling.

Beyond microbial remodeling, Se intake may reinforce gut homeostasis through molecular and metabolic pathways (Figure 2). By enhancing the expression of key intestinal selenoproteins, including GPX2 and GPX4, which maintain mucosal redox balance in the intestine, and endoplasmic reticulum resident selenoproteins that alleviate endoplasmic reticulum stress, Se further shapes gut microbial structure and function [54,55]. Particularly TrxR, dietary Se converts pro-pathogenic tetrathionate into benign thiosulfate, effectively restricting opportunistic pathogens like *Salmonella* that exploit inflammatory niches [56]. These reductive shifts, coupled with Se-mediated suppression of NF-κB signaling, alleviate inflammation, oxidative stress and epithelial barrier damage [57]. Consequently, compromised commensal communities (e.g., depleted *Lactobacillus* and *Bacteroides*) regain diversity, while butyrate-producing *Clostridia* further activate anti-inflammatory PPARγ pathways [58]. Notably, Se yeast particularly increases the content of butyric acid which regulates the repair of the intestinal mucus barrier, promotes the production of IL-10, and suppresses neutrophil effector functions [45,59,60,61]. This restructured microbiota reciprocally amplifies host selenoprotein synthesis, establishing a self-sustaining cycle that resolves inflammation and promotes mucosal repair. Thus, dietary Se supplementation acts at the microbiota–immune interface to break pathogenic feedback loops characteristic of chronic gut disorders. In recent years, engineered selenium-based formulations have attracted increasing attention for their potential to regulate intestinal homeostasis. The representative product is Se-enriched probiotics [62,63]. For instance, a *Lactobacillus casei* with chimeric Se dots designed by Chinese researchers can effectively improve the acid resistance and intestinal adhesion of this probiotic, achieving the restoration of gut redox and microbiota homeostasis, and significantly improving the symptoms of ulcerative colitis in multiple mouse models and a cynomolgus monkey model. In addition, some natural or synthetic Se polysaccharides and Se nanoparticles decorated with polysaccharides have also been reported to significantly reduce the abundance of pathogenic bacteria (e.g., *Campylobacter* and *Ruminococcaceae*) in inflammatory bowel disease, restoring intestinal homeostasis [64,65]. In conclusion, the Se–gut microbiota axis is a complex and interactive system in gut health. Further research in this area will help us better understand the mechanisms involved and develop more effective strategies for promoting gut health.

## 4. Se–Gut Microbiota Axis in Distal Tissue Health

Se–gut microbiota axis, as an important hub of body homeostasis regulation, coordinates the physiological balance of distal organs (such as liver, muscle, brain and kidney) through the synergistic interaction between gut microbiota metabolites and host regulatory signaling networks, and realizes the comprehensive regulation of stress defense, metabolic reprogramming and tissue biofunction maintenance. Among these interorgan communication systems, the gut–liver axis is well documented, with abundant evidence from both clinical and preclinical studies. By comparison, the gut–brain, gut–muscle, gut–kidney and gut–gonadal axes are largely conceptual frameworks established based on preclinical findings. Their physiological roles and clinical implications still await further validation.

### 4.1. Se and Gut–Liver Axis

The Se–gut–liver axis represents a critical regulatory center that links dietary Se intake to hepatic homeostasis via gut microbial metabolites and immune modulation. Disruption of the intestinal barrier, often resulting from gut dysbiosis, allows lipopolysaccharide (LPS) to translocate into the portal circulation and trigger hepatic inflammation via the TLR4/NF-κB pathway. Dietary Se counteracts this inflammatory axis at multiple levels. It has been found that Se could modulate gut microbiota composition and reduce the excessive abundance of LPS-enriched Gram-negative taxa, including *Escherichia-Shigella* in cadmium-exposed mice [65,66]. This modulation reduces the LPS load entering the portal circulation at the source. In parallel, Se directly intervenes in hepatic inflammatory signaling by upregulating selenoprotein including GPX and TrxR [67]. Even when small amounts of LPS circumvent the intestinal barrier and reach the liver, this Se-dependent antioxidant system effectively suppresses excessive activation of the TLR4/NF-κB pathway and attenuates hepatocyte inflammatory responses [68,69]. This triple action, barrier reinforcement, microbial remodeling, and direct immune modulation, positions Se as a critical regulator of the gut–liver inflammation axis.

These gut-derived substances can even disrupt hepatic metabolism, leading to various liver diseases, including non-alcoholic fatty liver disease (NAFLD), acute liver injury, (ALI), liver fibrosis, and even hepatocellular carcinoma [70,71]. NAFLD, the hepatic manifestation of metabolic syndrome, has become a leading cause of chronic liver disease in many regions worldwide, and now affects approximately 38% of the global adult population [72,73]. A study in a high-fat diet (HFD)-induced NAFLD mouse model has proven that supplementation with low-molecular-weight chitosan-stabilized Se nanoparticles (LCS-SeNPs) significantly alleviates hepatic steatosis and metabolic abnormalities [74]. Mechanistically, LCS-SeNPs reshape the gut microbiota by increasing the relative abundance of genera such as *Akkermansia*, *Bifidobacterium*, and *Muribaculaceae*, while suppressing obesity-associated harmful bacteria including *Anaerotruncus*, *Lachnoclostridium*, and *Proteus* [74]. These microbial shifts are accompanied by reduced hepatic lipid accumulation, lower serum ALT/AST levels, and improved glucose tolerance. It demonstrates that Se-driven microbiota modulation may effectively mitigate NAFLD progression. Despite its beneficial effects, the impact of Se on NAFLD is subject to a U-shaped dose–response curve. Some research shows an inverse association between serum Se and NAFLD risk, while others, particularly in Chinese populations, link higher plasma Se with increased NAFLD prevalence [75,76]. Experimental evidence suggests that excessive Se may exacerbate hepatic insulin resistance via oxidative stress-mediated JNK activation and disrupt glucose homeostasis in a nongenetic mouse model of type 2 diabetes [77]. This narrow therapeutic window necessitates precise dose optimization in any Se-based intervention for NAFLD, and underscores the need for personalized approaches.

ALI, often induced by toxins or drugs, is exacerbated by gut-derived endotoxemia [78]. In a carbon tetrachloride (CCl_4_)-induced ALI mouse model, dietary supplementation with Se-enriched *Lactobacillus* plantarum selectively boosts the abundance of SCFA-producing bacteria, including lactate-producing *Lactobacillus*, butyrate-producing *Butyricicoccus* and *Clostridiales*, and acetate/propionate-producing *Phaseolaretobacterium* [79]. Though SCFAs are not uniformly protective and their functions depend on concentration, action site, cell type, and inflammatory status, appropriate elevated SCFAs, particularly butyrate, exert hepatoprotective effects by activating G-protein-coupled receptors (GPR41/43) on intestinal epithelial cells, reinforcing the gut barrier and reducing systemic inflammation [80]. Notably, this intervention does not broadly restructure the microbiota but rather amplifies key functional groups, highlighting the precision of Se-mediated microbial modulation.

The regulatory influence of Se on liver health extends beyond inflammation control to direct modulation of hepatic metabolism. This process is largely mediated by gut microbial metabolites including short-chain fatty acids (SCFAs) and bile acids (BAs), which serve as vital signaling molecules bridging gut microbial activity and liver function. SCFAs (acetate, propionate, butyrate) produced by Se-enriched microbiota enter the portal circulation have been reported to directly influence hepatic lipid and glucose metabolism in a context-dependent fashion. In hepatocytes, butyrate is capable of activating AMPK and suppressing histone deacetylases (HDACs), which facilitates fatty acid oxidation and restrains de novo lipogenesis (DNL) [81]. Accumulating mechanistic evidence also indicates that butyrate modulates LKB1 and INSIG signaling pathways, an effect associated with the mitigation of hepatic steatosis [82]. Additionally, SCFAs stimulate intestinal enteroendocrine cells to secrete glucagon-like peptide-1 (GLP-1) and peptide YY (PYY), which enhance insulin sensitivity and reduce hepatic glucose output [83].

BAs represent a parallel pathway through which Se modulates liver metabolism via gut microbiota. Synthesized from cholesterol in the liver, primary BAs are modified by gut microbiota via deconjugation and 7α-dehydroxylation to form secondary BAs [84]. As documented in previous studies, secondary BAs are capable of activating the nuclear receptor farnesoid X receptor (FXR) and membrane receptor Takeda G-protein-coupled receptor 5 (TGR5) [85,86]. Se supplementation could reshape the gut microbial community to sustain BA homeostasis. This homeostatic state appears to directly regulate hepatic cholesterol metabolism and acts as a key physiological node that helps protect against liver injury [87]. For example, marine-derived Se-enriched chondroitin sulfate (CSSE) has been shown to elevate the abundance of the butyrate-producing genus *Faecalibaculum*, which facilitates intestinal FGF19 secretion [88]. FGF19 travels to the liver, where it binds to FGFR4/β-klotho complexes and tends to suppress CYP7A1 expression, thereby lowering BA synthesis and cholesterol accumulation [89]. Simultaneously, secondary BAs may activate hepatic FXR, which in turn upregulates the expression of small heterodimer partner (SHP). This cascade may further restrain CYP7A1, the rate-limiting enzyme for hepatic BA synthesis, and promotes fatty acid oxidation [86]. Additionally, in an NAFLD mouse model established using a Western diet plus CCl_4_, organic selenium compounds (L-selenocystine, L-selenomethionine, and L-selenomethylcysteine) were found to downregulate CYP7A1 while upregulating FXR expression, accompanied by a marked decline in total BA levels [90]. Notably, it has been reported that different Se forms and doses exert divergent impacts on BA metabolism alongside gut microbiota modulation. For instance, in mice models, low-dose Na_2_SeO_3_ primarily disrupts hepatic BA homeostasis via suppressing hepatic FXR activity, causing intrahepatic BA accumulation and elevated Firmicutes/Bacteroidetes ratio, low-dose nano-Se mainly reduce Firmicutes/Bacteroidetes ratio rather than altering circulating BA levels, while moderate-dose nano-Se triggers obvious gut dysbiosis and pathogenic bacteria proliferation, presenting toxic effects [52].

### 4.2. Se and Gut–Brain Axis

Serving as a pivotal hub for systemic homeostasis regulation, the Se–gut microbiota axis may extend its influence to the central nervous system, forming the microbiota–gut–brain axis. This axis plays a central role in maintaining neural homeostasis and counteracting neurological damage through multiple synergistic mechanisms. Systemic chronic inflammation acts as a common pathological hub driving a range of brain disorders, from neurodegenerative diseases to mood disorders, by disrupting the blood–brain barrier, activating microglia, and triggering neurotoxic cascades [91]. Se supplementation can effectively mitigate the development and progression of these related brain pathologies. Studies have shown that Se deficiency promotes gut dysbiosis and increases LPS translocation, which disrupts blood–brain barrier integrity via the TLR4/NF-κB pathway and facilitates neurotoxin and inflammatory mediator entry into brain tissue [39,92]. In contrast, adequate Se restores gut homeostasis, reduces inflammation, and preserves cognitive function [93].

The regulatory role of the Se–microbiota–brain metabolite axis has also been validated in various neurological disease models. In an Alzheimer’s disease (AD) mice model, Se nanoparticles derived from Se-enriched *Lactobacillus casei* modulated the abundance of *Gordonibacter*, downregulated cerebral Aβ deposition and Tau protein hyperphosphorylation, and elevated brain-derived neurotrophic factor levels, thereby improving cognitive function [94]. Similarly, in Parkinson’s disease (PD) mice models, excessive SCFAs derived from the gut microbiota activate microglia in the substantia nigra and striatum of the brain, upregulating the expression of pro-inflammatory factors TNF-α and IL-6 [95]. This promotes abnormal aggregation of alpha-synuclein and enhances neuroinflammation, ultimately worsening motor dysfunction [96]. However, low-dose SCFAs typically activate pathways such as AMPK and Nrf2 to exert protective effects [97]. Remarkably, Se supplementation can reshape gut microbiota, and alleviate these pathological changes, but this protective effect is still in the animal model research stage and lacks human clinical evidence to confirm these effects.

Beyond its anti-inflammatory effects, the Se–gut–brain axis directly influences the synthesis of key neurotransmitters that regulate mood and cognition. Gut-derived serotonin may influence the brain indirectly through neural, immune, or metabolic pathways [98]. It is primarily involved in regulating mood stability, sleep–wake cycles, and appetite, and its dysfunction is clearly associated with brain health risks such as depression, anxiety disorders, and insomnia [99,100,101]. Se modulates specific commensal bacteria such as certain *Clostridia*, fermenting dietary fiber to produce SCFAs, which can directly stimulate intestinal enterochromaffin cells (ECs) to upregulate the expression of the key rate-limiting enzyme (tryptophan hydroxylase 1(TPH1)) for serotonin synthesis, and, as a result, significantly promoting serotonin production [102]. Secondly, through their metabolic activities, microbes influence the local immune environment, thereby regulating the metabolic fate of tryptophan to favor serotonin synthesis over alternative pathways [103]. Unlike serotonin, γ-aminobutyric acid (GABA), the primary inhibitory neurotransmitter in the central nervous system, is regulated by gut microbiota through a more direct biosynthetic mechanism [104]. Under the modulation of Se, certain strains of gut bacteria express glutamate decarboxylase (GAD) and can directly convert glutamate into GABA within the gut [105]. Although GABA itself does not readily cross the blood–brain barrier, its accumulation in the intestine can modulate anxiety, stress responses, and sleep homeostasis by acting on GABA receptors in the enteric nervous system and vagus nerve, consequently relaying inhibitory signals to the brain [106]. These Se-modulated neurotransmitter pathways contribute to the axis’s protective effects against not only neurodegenerative diseases but also mood disorders such as depression and anxiety.

### 4.3. Se and Gut-Other Tissues Axis

In addition to affecting liver and brain tissue, the selenium gut microbiota axis can also affect the health of other tissues, including skeletal muscle, kidneys, etc.

The concept of a gut–muscle axis has emerged from growing evidence that intestinal microbiota influence muscle physiology through circulating microbial metabolites. Gut microbiota-derived metabolites can regulate skeletal muscle homeostasis through multiple mechanisms involving inflammation and immune modulation, energy metabolism, endocrine signaling, and insulin sensitivity [107]. Dietary Se integrates into this axis by shaping the microbial community and metabolite composition, and this Se–gut–muscle axis serves as a critical regulatory hub linking Se intake to peripheral muscle homeostasis. In models of glucocorticoid-induced muscle atrophy, Se supplementation not only mitigates muscle wasting but also restructures the microbial community, increasing the abundance of SCFA-producing bacteria, particularly the *Lactobacillus* and *Dubosiella*, [108]. Meanwhile, in aged telomere-humanized male mice, dietary Se deficiency can specifically accelerate the enrichment of *Akkermania muciniphila* in the intestinal flora and type 2 diabetes, and compensatory increases the content of SCFAs [109]. Beyond producing metabolites to directly regulate skeletal muscle health, certain gut bacteria may directly influence Se availability to muscle tissue. The abundance of *Prevotella copri* and the levels of branched-chain amino acids (BCAAs) are significantly reduced in the intestinal tract of patients with sarcopenia, which may support its potential value for interventions against sarcopenia [110]. Interestingly, previous studies have indicated that this species is positively correlated with muscle Se content in pigs, and supplementation with this bacterium can promote selenium accumulation in tissues [111].

The kidney is among the organs with the highest Se concentrations in the body, underscoring its strong dependence on selenoproteins for antioxidant defense and redox homeostasis [112]. Chronic kidney disease (CKD), which affects nearly 10% of the global population, disrupts this balance through a vicious cycle driven in part by gut microbiota-derived uremic toxins, thereby aggravating renal dysfunction and disease progression [113,114]. Through modulation of gut microbial ecology and their metabolites, dietary Se can influence renal function and susceptibility to injury, highlighting the emerging importance of the Se–gut–kidney axis in kidney health and disease.

Gut-derived uremic toxins as drivers of renal Se dysregulation. It may induce profound alterations in gut microbiota composition, leading to the systemic accumulation of microbial metabolites that impair organ function [115]. Among the more than 90 uremic solutes, indoxyl sulfate (IS) is one of the most extensively studied solutes, mainly derived from bacterial metabolism of dietary tryptophan in the intestine. It can be sulfated by the liver and secreted by the proximal tubules through organic anion transporters [116]. Mechanistically, IS induces hepatocyte oxidative stress, leading to dose- and time-dependent downregulation of SELENOP [117,118,119]. As the primary plasma Se transporter, impaired SELENOP synthesis restricts Se delivery to the kidney, consequently depleting renal GPX4 and promoting ferroptotic tubular injury [120,121,122]. This establishes a direct mechanistic link between renal Se depletion, microbial tryptophan metabolism, and loss of tubular integrity.

Se intervention counteracts toxin-induced renal injury. Dietary Se supplementation has demonstrated protective effects against nephrotoxicity in multiple experimental models. In piglets exposed to ochratoxin A (OTA), Se-enriched probiotics significantly attenuates OTA-induced renal injury [123]. In a mouse model of cadmium-induced renal toxicity, co-administration of Lactobacillus casei-derived SeNPs with cadmium reduce pro-apoptotic Bax and increase anti-apoptotic Bcl-2 expression, while decreasing renal inflammatory markers (TNF-α, IL-6, NF-κB) in a dose-dependent manner [124]. Similarly, Se-enriched probiotics have shown efficacy against drug-induced nephrotoxicity. Cyclosporin A (CsA), an immunosuppressant used in ulcerative colitis treatment, is limited by dose-dependent nephrotoxicity [125]. In both colitis and CKD mouse models, co-administration of Se-enriched *Bifidobacterium* longum DD98 with CsA significantly attenuated CsA-induced kidney injury, reducing serum creatinine and blood urea nitrogen levels while preserving renal architecture [126]. This nephroprotective effect was accompanied by favorable shifts in gut microbiota composition, including increased alpha diversity and enrichment of beneficial taxa such as *Ruminococcaceae_UCG-014* and *Lachnospiraceae_NK4A136* [126].

Similarly, the reproductive organs also exhibit special sensitivity to Se status due to their dependence on redox balance for gametogenesis, leading to the concept of a Se–gut–gonadal axis [127]. This regulatory axis exerts systemic and coordinated effects on the testes or ovaries via the circulatory system, modulating redox homeostasis, energy metabolism, and hormonal balance, ultimately leading to improved reproductive health outcomes. In male models, the dose and form of Se are critical for activating this axis. Rooster studies indicate a narrow effective window (0.25–0.35 mg/kg), with nano-Se demonstrating superior efficacy over conventional forms in improving sperm motility due to their higher bioavailability [128]. Rat experiments further reveal that low-dose selenized glucose (SeGlu, 0.15 mg Se/L) optimally enhances sperm quality [129]. This improvement is accompanied by significant remodeling of the gut microbiota, marked by an increase in beneficial SCFA-producing genera such as *Intestinimonas*, *Christensenella*, *Butyrivibrio*, and *Roseburia*, alongside suppression of potentially harmful bacteria like *Rikenella* [129]. This Se-optimized microbial structure systematically improves the distal testicular microenvironment through microbial metabolites (e.g., butyrate) and immunomodulatory effects, including enhanced antioxidant capacity (elevated SOD and GPx activities), regulation of local metabolism and hormone balance, and ultimately, promotion of seminiferous epithelial development and spermatogenesis. The protective role of this axis is particularly evident under environmental stress. In mice exposed to a chemical cocktail of heavy metals and drugs, testicular metabolomics showed severe disruption. Se supplementation remodeled the correlation network between gut microbiota and testicular metabolites, reversed dysregulation of key signaling molecules such as anandamide and prostaglandin metabolites, and restored perturbed pathways including glycerolipid metabolism and glutathione metabolism toward normal states, thereby mitigating reproductive toxicity [130].

Notably, this regulatory axis also applies to the female reproductive system. Studies in laying hen models have found that organic Se or nano-Se supplementation is more effective than inorganic Se in improving egg production and ovarian antioxidant capacity [131]. The mechanism is similarly linked to gut microbiota modulation. These Se sources increase the abundance of beneficial genera such as *Prevotella* and *Olsenella* in the cecum while synchronously regulating ovarian pathways including PPAR signaling, linoleic acid metabolism, and glycerophospholipid metabolism, supporting follicular development and function through improved energy utilization and membrane integrity [131]. Finally, a key mouse study employing antibiotic-mediated gut microbiota depletion directly demonstrated the necessity of the microbiota in this axis. In the absence of gut bacteria, Se supplementation could elevate total testicular Se content but failed to induce the normal expression profile of selenoproteins (e.g., GPx); moreover, its protective effect against testicular structural damage is substantially diminished [132]. This confirms that an intact gut microbial ecosystem is a prerequisite for Se to exert its reproductive protective functions.

## 5. Conclusions

The well-established Se–gut–tissue axis provides a critical framework for understanding dietary the core mechanisms of Se in host regulation. Research demonstrates that Se (particularly organic or new synthetic forms) may reshape gut microbiota structure and function through bidirectional interactions with gut bacteria. This dual mechanism not only enhances bioavailability by modulating microbial composition, but also enables metabolic byproducts to act as signaling carriers, transmitting the biological effects of Se to distal tissues including the liver, muscles, brain and kidneys.

**Figure 3:**
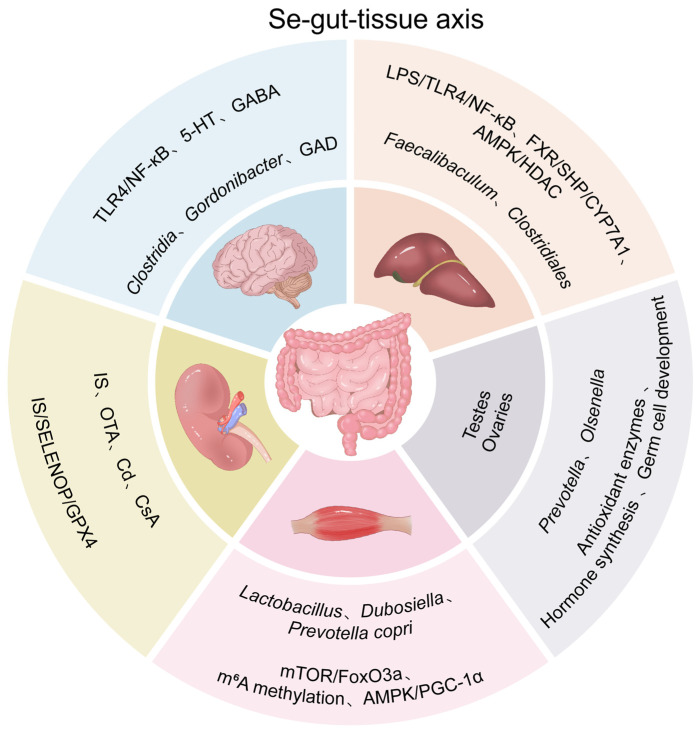
Mechanisms of selenium-supported extra-intestinal health via microbiota remodeling.

This regulatory process depends on the cross-regulation and synergy of key signaling pathways and maintains the stability of multiple tissues in the body through multiple pathways such as antioxidant stress, inhibition of inflammatory response, enhancement of barrier function and metabolic reprogramming (Figure 3). However, there are still major challenges in this research:

1. Causality and translation: Most evidence is from animal models and cannot be directly extrapolated to humans. The causal relationship between Se and microbiota needs to be verified through sterile models and fecal transplantation; microbiota changes may be secondary to metabolic effects rather than causal.
2. Dose and safety: Se has a clear U-shaped dose–response and high Se causes selenosis and may increase metabolic disease risk.
3. Methodology: 16S rRNA sequencing has limited resolution and functional information and data are compositional and confounded by diet/environment.
4. Evidence hierarchy: Microbiota–disease correlations, animal interventions, enzymatic data, and hypotheses are mixed, so reliability levels are not clearly stratified.
5. Translation barriers: Advanced Se delivery systems (nanoformulations, Se-probiotics, Se-prebiotic complexes) face nanotoxicity, stability, inter-individual variability, and lack of long-term human safety data.

Future precise nutrition should consider administration Se forms, and clinical translation must address nanotoxicity and stability; dose-dependent responses and species/individual differences require precise intervention strategies, and most evidence is from animal models that cannot be directly extrapolated to humans; and the underlying mechanisms of specific strains, metabolites and molecular targets need to be integrated by multi-omics research. Therefore, efforts should be made to develop advanced Se delivery systems (such as Se–prebiotic complexes), explore cross-tissue axis interaction mechanisms, and verify the therapeutic potential in disease models (such as metabolic disorders, neurodegenerative diseases).

## Data Availability

The original contributions presented in this study are included in the article. Further inquiries can be directed to the corresponding author(s).
